# Acceptability of a Clinician-Assisted Computerized Psychological Intervention for Comorbid Mental Health and Substance Use Problems: Treatment Adherence Data from a Randomized Controlled Trial

**DOI:** 10.2196/jmir.1522

**Published:** 2011-01-27

**Authors:** Frances Kay-Lambkin, Amanda Baker, Terry Lewin, Vaughan Carr

**Affiliations:** ^3^School of PsychiatryUniversity of New South WalesSydneyAustralia; ^2^Centre for Brain and Mental Health ResearchUniversity of NewcastleCallaghanAustralia; ^1^National Drug and Alcohol Research CentreUniversity of New South WalesRandwick, NSWAustralia

**Keywords:** computerized cognitive behavior therapy, brief intervention, comorbidity, depression, alcohol use problems

## Abstract

**Background:**

Computer-delivered psychological treatments have great potential, particularly for individuals who cannot access traditional approaches. Little is known about the acceptability of computer-delivered treatment, especially among those with comorbid mental health and substance use problems.

**Objective:**

The objective of our study was to assess the acceptability of a clinician-assisted computer-based (CAC) psychological treatment (delivered on DVD in a clinic-setting) for comorbid depression and alcohol or cannabis use problems relative to a therapist-delivered equivalent and a brief intervention control.

**Methods:**

We compared treatment acceptability, in terms of treatment dropout/participation and therapeutic alliance, of therapist-delivered versus CAC psychological treatment. We randomly assigned 97 participants with current depression and problematic alcohol/cannabis use to three conditions: brief intervention (BI, one individual session delivered face to face), therapist-delivered (one initial face-to-face session plus 9 individual sessions delivered by a therapist), and CAC interventions (one initial face-to-face session plus 9 individual CAC sessions). Randomization occurred following baseline and provision of the initial session, and therapeutic alliance ratings were obtained from participants following completion of the initial session, and at sessions 5 and 10 among the therapist-delivered and CAC conditions.

**Results:**

Treatment retention and attendance rates were equal between therapist-delivered and CAC conditions, with 51% (34/67) completing all 10 treatment sessions. No significant differences existed between participants in therapist-delivered and CAC conditions at any point in therapy on the majority of therapeutic alliance subscales. However, relative to therapist-delivered treatment, the subscale of Client Initiative was rated significantly higher among participants allocated to the BI (F_2,54_ = 4.86, *P* = .01) and CAC participants after session 5 (F_1,29_ = 9.24, *P* = .005), and this domain was related to better alcohol outcomes. Linear regression modeled therapeutic alliance over all sessions, with treatment allocation, retention, other demographic factors, and baseline symptoms exhibiting no predictive value.

**Conclusions:**

Participants in a trial of CAC versus therapist-delivered treatment were equally able to engage, bond, and commit to treatment, despite comorbidity typically being associated with increased treatment dropout, problematic engagement, and complexities in treatment planning. The extent to which a client feels that they are directing therapy (Client initiative) may be an important component of change in BI and CAC intervention, especially for hazardous alcohol use.

**Trial Registration:**

Australian New Zealand Clinical Trials Registry ACTRN12607000437460; http://www.anzctr.org.au/trial_view.aspx?ID=82228 (Archived by WebCite at http://www.webcitation.org/5ubuRsULu)

## Introduction

Although mental health problems are highly prevalent, the gap between need for effective treatment and treatment received is large, particularly for counseling interventions [1]. The World Health Organization reported that this gap is 56% for depression and 78% for alcohol abuse and dependence [2,3]. Comorbidity, or the co-occurrence of two or more disorders such as depression and alcohol abuse/dependence, is the rule rather than the exception in clinical practice [4,5] and compounds the difficulties in treatment access [6].

Comorbidity has largely been ignored in research and policy, especially depression and alcohol/other drug (AOD) use comorbidity, and treatment services do not generally provide well for people with multiple disorders [7]. Many general practitioners and specialist clinicians lack the confidence or skills to screen and assist patients with comorbid mental disorders and AOD use problems, and clients are often reluctant to discuss these issues with their health care providers [8]. As a result, treatment may not be accessed until the problem is severe, if at all. Thus, improving access to effective treatments for high-prevalence, treatable disorders such as depression and AOD use is an important health care priority.

Brief interventions (BIs) have been widely implemented in the AOD field with a view to extending the reach of interventions, especially for alcohol problems [eg, 9]. It has been suggested that BIs are most appropriate for people with less severe drinking problems and are best combined with more intensive, longer treatments for people with moderate to severe problems [10]. Accumulating evidence supports the effectiveness of BIs for people with comorbid depression and AOD use problems [eg, 11-13].

The increased availability and use of computerized or internet-based programs as a supplement to health care is also a potential solution to accessibility problems [14]. A recent systematic review of e-therapy for mental health problems identified 14 randomized controlled trials supporting the efficacy of computer- or internet-based treatments for depression, panic disorder, chronic tension/migraine, trauma, insomnia, obesity, complicated grief, and eating disorder [3]. This mode of delivery is also supported by a recent randomized controlled trial of internet-based self-help for alcohol use problems [15].

We recently reported the results of the (to our knowledge) first randomized controlled trial of clinician-assisted computer-based (CAC) psychological treatment for depression and AOD use comorbidity [16]. Therapist-delivered treatment was directly compared with a BI and CAC treatment. BI was shown to be beneficial for problem drinking among this depressed sample over the short term. No significant differences were found between the CAC and therapist-delivered treatment modalities, with significant improvement across a range of depression, AOD, and quality-of-life outcomes at the 12-month follow-up assessment. Therapist and CAC treatments produced effect size differences in depression and functioning of greater than 0.25 standard deviations relative to the BI at 12-month follow-up. The BI and CAC intervention were associated with moderate to large effect sizes for alcohol consumption at 12 months, with CAC participants reporting significantly better overall substance use outcomes than the other conditions, and were five times more likely than BI participants to report a 50% reduction in hazardous substance use days [16]. Intention-to-treat analyses confirmed each of the above findings. Clinician assistance provided in the computer condition was on average 12.5 minutes of generic contact per session (eg, compliance checking, mood, and AOD use assessment).

A central component in the uptake and success of any treatment is acceptability to patients, particularly when translating results from clinical trials to clinical practice. This is especially relevant for different modes of treatment delivery, such as computerized therapy, which offers alternatives to traditional, face-to-face treatment. However, a recent review reported that very little attention has been paid to the acceptability of computerized psychological treatment, notably cognitive behavior therapy (CBT), compared with traditional approaches [17]. This is also true of the BI literature. Treatment acceptance, or readiness to accept help, may be the determining factor in whether or not clients make changes to their life circumstances. 

The present study aims to address this gap, by reporting on the acceptability of CAC CBT for comorbid depression and AOD use problems relative to an equivalent therapist-delivered CBT treatment and BI. As suggested by Kaltenthaler and colleagues [17], proxy criteria for patient acceptability of treatment include treatment participation and retention, and questionnaires or surveys that cover patient acceptability or satisfaction with treatment. In this study, treatment attendance and patient-rated therapeutic alliance throughout the treatment period were used as proxies for treatment acceptability, and these indices were compared for therapist-delivered versus CAC treatment. Therapeutic alliance associated with BI is also reported.

## Methods

The methods and study design have been reported in detail elsewhere [16]. Eligibility criteria were as follows: (1) current depressive symptoms (score of 17 or greater on the Beck Depression Inventory, BDI-II [18]), (2) current problematic use of alcohol (ie, consumption above recommended drinking levels as suggested by the National Health and Medical Research Council of Australia) or cannabis (at least weekly use), (3) absence of a brain injury, organic brain disease, and/or significant cognitive impairment, and (4) ability to understand English.

Participants were recruited across New South Wales, Australia. Referral to the project was via a range of sources, most commonly via self-referral in response to television interviews conducted with the investigators (39/97, 40%), or newspaper articles promoting the study (53/97, 55%). A comparatively small proportion of participants were recruited via local mental health outpatient clinics (3/97, 3%) and AOD outpatient services (2/97, 2%). Following initial assessment, participants received one face-to-face session with a therapist comprising feedback, case formulation, and initial goal setting. Upon completion of this session, participants were randomized to no further treatment (BI only), nine weekly sessions of combination CBT and motivational interviewing (MI) delivered exclusively by a therapist, or nine sessions of CAC CBT/MI with weekly brief check sessions (approximately 12.5 minutes) delivered face to face by a therapist. Check-in sessions were generic in nature, comprising a check to ensure completion of the module, review of homework set for the coming week, and a mood/AOD assessment. The computerized component of CAC was DVD-based, and delivered via computers located at the study clinics. The DVD program was text-based, with interactive components including video vignettes, printable worksheets and handouts, and options for tailoring content to the participant’s stage of change or area of need. All text contained in the CAC intervention was presented by a voiceover to accommodate people with reading difficulties. Follow-up occurred 3, 6, and 12 months following baseline. Three-month (posttreatment) outcomes are reported here because of their temporal proximity to the treatment attendance and alliance indices.

### Measures

The following instruments are relevant to the analyses reported below:

1. *Demographic information*: using subscales of the Diagnostic Interview for Psychosis (DIP) [19], basic demographic information was collected (including age and gender).
2. *BDI-II* [18]: a 21-item self-report questionnaire screening for the presence of depressive symptoms over the previous 2-week period. Items cover the range of symptoms listed in the Diagnostic and Statistical Manual of Mental Disorders, 4th revision [20] for major depressive disorders. The questionnaire has been validated with adult and adolescent populations, and is used to screen for depressive symptoms among people with AOD use problems [21].
3. *Opiate Treatment Index* (OTI [22]: addresses the quantity and frequency of use across 11 substances, including alcohol and cannabis. Each drug type is assessed individually, and clients report on their last three using occasions in the month prior to assessment, estimating the amount of drug consumed on each of these occasions. An average use index for the previous month is calculated for each drug.
4. *Hazardous Use Index*: an aggregate global AOD use score was calculated for all participants that estimated, using the OTI, the number of day equivalents in the previous 28-day period that participants used a range of 10 drug types at harmful levels (range 0-280).
5. *Beck Hopelessness Scale* (BHS) [23]: a 20-item self-report instrument that measures optimism about the future and indirectly estimates suicide risk. Participants complete the scale by providing true/false responses to 20 statements related to their thoughts about the future over the previous 2-week period.
6. *Readiness to Change* [24]: a questionnaire based on the stage-of-change model [25]. Participants completed one questionnaire for each drug they were using at baseline (alcohol, cannabis) and rated their agreement with 15 statements relating to their baseline AOD use according to a 5-point Likert scale (1 = strongly disagree, 5 = strongly agree). The scale is divided into three subsections that relate to the following stages of change: precontemplation, contemplation, and action. Scores are totaled for the items particular to each subsection, and the subsection with the highest total score is the baseline stage of change for that drug. For the purposes of this analysis, stage of change was dichotomized into precontemplation versus not (ie, contemplation or action).
7. *Agnew-Davies Relationship Measure* (ARM) [26]: a measure of therapeutic alliance containing 28 self-report items regarding client- and therapist-based domains and impressions of the client-therapist relationship. Each item is rated according to a 7-point Likert scale, with higher scores indicating more positive perceptions of alliance. Five subscales are derived from item ratings [26]: (1) Bond, which represents the friendliness, acceptance, and understanding felt by the client in the therapeutic relationship (eg, “I feel accepted in therapy”, “I feel friendly toward my therapist”), (2) Partnership, which concerns the extent to which the client feels that he or she is working jointly on therapeutic tasks with the therapist (eg, “my therapist follows his or her own plans”, “my therapist and I agree about how to work together”), (3) Confidence, which concerns the extent of optimism and respect for the therapy in which the client is engaged (eg, “I feel critical of or disappointed in my therapy”, “I feel optimistic about my progress in therapy”), (4) Client Initiative, which examines how well the client takes responsibility for the direction of therapy (eg, “I take the lead when I’m in therapy”, “I am expected to take responsibility rather than be dependent on therapy”, “I look to my therapist for solutions to my problems”), and (5) Openness, which concerns the extent to which a client feels free to disclose personal issues and worries in therapy (eg, “I can discuss personal matters I am ordinarily ashamed or afraid to reveal”, “I am worried about embarrassing myself in therapy”).
8. *Treatment attendance*: A record of attendance was kept for each participant to determine the number of treatment sessions they attended during the course of therapy. The maximum possible attendance for participants in the BI was 1, with CAC and therapist-delivered participants having access to a maximum of 10 sessions.

### Statistical Analysis

Data were analyzed using the Statistical Package for Social Sciences version 17.0 (SPSS Inc, Chicago, IL, USA). 

### Baseline Characteristics

Exploratory data analysis was performed on all measures relevant to the current study.

### Treatment Attendance and Follow-up Participation

Chi-square analysis examined the proportion of treatment sessions attended (full complement vs not) for therapist-delivered and CAC condition participants. One-way analysis of variance (ANOVA) was used to examine the average attendance for the active treatment groups. For therapist-delivered and CAC condition participants, a dichotomous variable was also created to indicate whether an adequate dose of treatment had been received (yes/no). An adequate dose of treatment was considered to be attendance at 6 or more of 10 possible sessions, given that this exposed them to the majority of CBT/MI strategies included in the treatment program. Chi-square analysis was used to compare CAC and therapist-delivered condition participants on this new variable. Chi-square analysis also compared participants who completed the 3-month follow-up assessment with those who did not on gender and treatment attendance at the required number of sessions, and one-way ANOVAs examined completers and noncompleters on age, baseline levels of depression, alcohol and cannabis use, and total scores on the ARM.

### Therapeutic Alliance

Four subscales were calculated from participant responses to the ARM (Bond, Confidence, Openness, and Client Initiative). A total score was also calculated for each session (1, 5, and 10). One-way ANOVA compared scores on these subscales and total scores at each administration with treatment allocation. Change scores were created, representing the change in ARM total scores between sessions 1 and 5, sessions 1 and 10, and sessions 5 and 10, with positive scores indicating an increase in therapeutic alliance. Data were substituted with a change score of 0 when participants did not provide alliance ratings at sessions 5 and 10. Changes in ARM total scores using these variables, according to treatment allocation, were examined using one-way ANOVAs, and only for participants allocated to the therapist-delivered or CAC conditions. Power calculations were performed on the outcomes of these analyses using G*Power (Version 1.3.2, Franz Faul, Universitat Kiel, Kiel, Germany).

An average alliance total score and subscale scores were calculated for each participant, comprising the average of available ratings for each subscale or total score (n = 55). Within this dataset, Pearson correlations examined associations between average therapeutic alliance total and subscale scores and changes in depression, alcohol use, cannabis use, and hazardous use indices at the 3-month assessment relative to baseline. One-way ANOVA examined associations between alliance total scores, gender, treatment allocation, and retention. Multiple linear regression was used to predict alliance total score, using a set of predictors that included either alcohol or cannabis use variables (baseline use and stage of change), and a range of symptom (BDI-II, BHS) and treatment (allocation, adequate treatment) variables. G*Power (version 1.3.2) was used to estimate the power associated with each linear regression.

## Results

Detailed descriptions of the sample at baseline have been reported elsewhere, along with the impact of the interventions on key symptoms over a 12-month follow-up period [16]. 

### Baseline Characteristics

Table 1 displays the baseline sample demographics and Table 2 the presenting symptoms relevant to the current analysis.

### Treatment Attendance and Follow-up Participation

As indicated in Table 2, 35 participants (36%) were randomized to therapist-delivered treatment, and 32 (33%) were allocated to the CAC condition, following the BI session. Only three therapist-delivered participants (9%) and one CAC participant (3%) failed to return for any additional sessions following randomization (χ^2^_1_ = 0.7, *P* = .40). In these active therapy conditions, 51% (34/67) of participants attended the full complement of 10 therapy sessions, including 54% (19/35) of therapist-delivered participants and 47% (15/32) within the CAC condition (χ^2^_1_ = 0.4, *P* = .54). Therapist-delivered and CAC condition participants attended an average of 7 of their allocated 10 sessions (mean(therapist) 7.4, mean(CAC) 6.9, *F*_1,66_ = 0.39, *P* = .53). Two-thirds (44/67) attended an adequate dose of therapy (6 or more sessions): 69% of therapist-delivered (24/35) and 63% (20/32) of CAC treatment participants (χ^2^_1_ = 0.3, *P* = .60).

**Table 1:** Baseline demographics of participants in a randomized controlled trial of clinician-assisted computerized cognitive behavior therapy for coexisting depression and alcohol/other drug use problems (N = 97)

	Participants	
	Mean	SD
Age (years)	35.37	10.21
Baseline levels of depression (BDI-II total score)^a^	31.93	9.55
Baseline levels of alcohol use (standard drinks/day)^b^	5.05	5.67
Baseline levels of cannabis use (use occasions/day)^b^	10.00	15.06
Hazardous alcohol/other drug use index^c^	40.34	18.21

^a^ Beck Depression Inventory II (BDI-II).

^b^ Opiate Treatment Index (OTI) q score.

^c^ Estimated day equivalents in the previous month that participants used a range of 10 drug types at harmful levels (range 0-280).

**Table 2:** Baseline presenting symptom profiles

		n	%
Males:females		45:52	46:54
**Allocated to treatment**			
	Brief intervention - control	30	31
	Therapist-delivered therapy	35	36
	Clinician-assisted computer-based therapy	32	33
**Alcohol status**			
	Abstinent	16	16
	Using - below threshold	29	30
	Using - above threshold	52	54
**Cannabis status**			
	Abstinent	27	28
	Using - below threshold	1	1
	Using - above threshold	69	71
**Stage of change –alcohol use**			
	Precontemplative	27	28
	Contemplative	34	35
	Action	20	21
	Maintenance/abstinent	16	16
**Stage of change – cannabis use**			
	Precontemplative	10	10
	Contemplative	39	40
	Action	21	22
	Maintenance/abstinent	27	28

Completion of follow-up assessments was 85% (82/97) for 3-month postbaseline, 81% at 6 months (79/97), and 85% (82/97) at 12 months. In total, 67 participants (69%) completed all phases of assessment (baseline, and 3,6, and 12 months), with no significant differences between treatment groups in follow-up participation (BI: 21/30, 70%; CAC: 23/32, 72%; therapist: 23/35, 66%; χ^2^_2_ = 0.7, *P* = .70). 

In addition, no significant differences existed between participants who completed the 3-month follow-up assessment versus those who did not in terms of age (F_1,96_ = 1.25, *P* = .27), gender (χ^2^_1_ = 0.3, *P* = .59), or attendance at the required number of treatment sessions (χ^2^_1_ = 1.9, *P* = .17). Completers and noncompleters were also not significantly different on baseline measures of depression (F_1,96_ = 0.46, *P* = .50), alcohol use (F_1,9__6_ = 1.46, *P* = .50), or cannabis use (F_1,96_ = 0.03, *P* = .86), or on the total scores of the ARM following session 1 (F_1,__54_ = 0.23, *P* = .63), session 5 (F_1,__29_ = 0.36, *P* = .56), or session 10 (F_1,__16_ = .10, *P* = .92).

### Therapeutic Alliance

Table 3 displays the mean and standard deviations for each of four subscales of the ARM.

**Table 3:** Mean subscale scores on the Agnew Relationship Measure (ARM) [26]^a^ for people participating in a study of treatment for coexisting depression and substance use disorders (and their treating clinician), according to treatment allocation^b^

		Subscales of the ARM				Total Score
		Confidence	Client Initiative	Openness	Bond	
		Mean (SD)	Mean (SD)	Mean (SD)	Mean (SD)	Mean (SD)
**Session 1**^c^						
	BI (17/31, 57%)	6.13 (0.65)	4.16 (0.90)	5.60 (1.12)	6.21 (0.81)	22.10 (2.64)
	Therapist (14/35, 40%)	6.10 (0.80)	3.13 (1.03)	5.44 (1.45)	6.29 (0.63)	20.96 (2.73)
	CAC^d^ (24/32, 75%)	6.14 (0.78)	3.52 (0.92)	5.36 (1.58)	6.64 (0.49)	21.66 (2.49)
**Session 5**^c^						
	Therapist (10/35, 29%)	6.26 (0.43)	3.95 (0.37)	5.58 (0.70)	6.45 (0.37)	22.24 (1.66)
	CAC^d^ (20/32, 63%)	6.10 (0.64)	4.60 (0.62)	5.54 (1.04)	6.55 (0.66)	22.78 (1.90)
**Session 10**^c^						
	Therapist (5/35, 14%)	6.51 (0.46)	4.05 (0.89)	5.44 (0.99)	6.80 (0.21)	22.80 (1.45)
	CAC^d^(12/32 38%)	6.18 (0.53)	4.69 (0.71)	6.03 (0.73)	6.56 (0.49)	23.47 (1.88)

^a^ Increasing scores indicate increasing levels of therapeutic alliance.

^b^ Brief intervention (BI) – control participants did not complete these measures across all assessments given their treatment program comprised one session only.

^c^ Rates of completion of the ARM at each session are provided as a proportion of the total number of participants allocated to each condition.

^d^ Clinician-assisted computer-based condition (CAC) - this included therapist assistance of approximately 10 minutes per session.

As indicated in Table 3, very few differences were evident in therapeutic alliance as a function of treatment modality. At the conclusion of session 1, participants in the BI rated themselves significantly more highly on Client Initiative than did participants allocated to the therapist-delivered condition (F_2,54_ = 4.86, *P* = .01), with no differences existing between the BI and CAC conditions. At session 5, participants in CAC treatment rated themselves significantly more highly on questions relating to Client Initiative than did their counterparts receiving therapist-delivered treatment (F_1,29_ = 9.24, *P* = .005). This difference had disappeared by session 10 (F_1,16_ = 2.48, *P* = .14). 

Change scores were calculated for the change in ARM total scores between sessions 1 and 5, 1 and 10, and 5 and 10 for participants allocated to the therapist-delivered and CAC conditions. Data for participants who provided alliance ratings at session 1 but did not provide ratings at any other timepoint were substituted with a change score of 0. On average, alliance scores increased over the treatment period (mean(1 vs 5) -1.01, SD 2.48, mean(1 vs 10) 0.92, SD 1.85, mean(5 vs 10) 0.04, SD 1.21). One-way ANOVAs indicated that no significant differences existed between therapist-delivered and CAC participants in the amount of change in alliance between sessions 1 and 5 (F_1,37_ = 0.02, *P* = .96) and sessions 1 and 10 (F_1,37_ = .13, *P* = .72), with both treatment groups reporting increases in alliance between sessions 1 and 5 (mean(therapist) 1.04, mean(CAC) = 1.00) and sessions 1 and 10 (mean(therapist) 0.78, mean(CAC) 1.00). No significant differences existed in the amount of change in alliance scores between sessions 5 and 10 according to treatment allocation (F_1,37_ = 1.29, *P* = .26); however, therapist-delivered participants reported a small decrease in alliance between these sessions, while CAC participants reported a small increase of the same magnitude (mean(therapist) -0.25, mean(CAC) 0.21). Parallel analyses were conducted, without substituting data for noncompleters, and provided the same pattern of results.

### Predicting therapeutic alliance

Of the total sample, 55 (57%) provided alliance ratings following session 1, 30 provided session 5 alliance ratings, and 17 provided session 10 alliance ratings. For sessions 5 and 10 alliance ratings, this corresponded to 45% (30/67) and 25% (17/67) of eligible participants allocated to either therapist-delivered or CAC treatment (see Table 3). Given the missing data associated with completing therapeutic alliance ratings at sessions 5 and 10, alliance ratings were averaged over the available timepoints to produce an average score for each participant on each subscale and the total ARM score, providing a dataset of 55 for this analysis. 

### Associations with therapeutic alliance

No significant correlations existed between any of the subscales of the ARM or the total alliance score and age, change in depression (BDI-II) scores, hopelessness (BHS) scores, and cannabis use between baseline and 3-month follow-up. This was also true for baseline levels of depression, hopelessness, and cannabis and alcohol use. A significant modest positive correlation existed between scores on the subscale of Client Initiative and change in alcohol use between baseline and 3-month follow-up (Pearson *r* = 0.21, *P* = .05), with reductions in alcohol use during this time being associated with improved alliance ratings on this subscale.

One-way ANOVAs indicated no significant differences in alliance total score and subscale ratings and gender, stage of change for alcohol use, stage of change for cannabis use, and whether participants attended an adequate number of treatment sessions. There was a trend for treatment allocation to be associated with the subscale of Client Initiative (F_2,__54_ = 4.07, *P* = .05, power = 0.90), with post hoc analysis indicating that ratings on this subscale were significantly higher for participants in CAC treatment than in the therapist-delivered intervention. Power to detect differences in alliance subscales and total scores was low to moderate, and of the order of 0.6 for Bond, 0.08 for Confidence, 0.05 for Openness, and 0.78 for the total score.

### Linear Regression Analysis: Modeling Therapeutic Alliance

Two linear regression models were used to predict the average alliance total score, using models that included either the alcohol or cannabis use variable, and a range of symptom and treatment variables. Predictor variables included baseline depression (BDI-II total score), hopelessness (BHS total score), cannabis or alcohol use at baseline (OTI score), and stage of change for alcohol/cannabis (precontemplation vs contemplation/action, or nonuse), treatment allocation, and whether adequate treatment was received (yes/no). This combination of predictors did not significantly predict alliance total scores in either the alcohol (F_6,__46_ = 0.60, *P* = .73, power = 0.29) or cannabis model (F_6,__38_ = 0.33, *P* = .92, power = 0.20). 

Given the associations between treatment allocation, change in alcohol use, and the subscale score for Client Initiative, a third linear regression model examined average Client Initiative scores, using the predictor variables of change in depression, change in hopelessness, change in alcohol use, treatment allocation, adequate treatment received, and baseline stage of change for alcohol use. This model did not significantly predict scores on the Client Initiative subscale (F_6__,__46_ = 0.86, *P* = .54). Power for this regression, calculated post hoc, was low at 0.34 (calculated using G*Power, version 1.3.2).

## Discussion

This study compared treatment acceptability, in terms of treatment dropout/participation and therapeutic alliance, of therapist-delivered versus CAC psychological treatment for comorbid depression and AOD use problems. Results indicated that both modes of treatment delivery were of equivalent acceptability to participants. This was also true for participants who received a BI. This suggests that people with comorbid depression and AOD use problems, despite the engagement, retention, and treatment difficulties characteristic of this population, can develop strong attachment with a computer-delivered treatment program and commitment to complete an adequate dose of treatment with minimal therapist input. These results are discussed in detail below.

### Treatment Attendance

All participants were randomly assigned to therapist-delivered versus CAC treatment following one face-to-face session. Take-up rates of both modes of treatment were high following randomization, with 91% (32/35) of therapist-delivered and 97% (31/32) of CAC treatment participants returning for at least one session. Over the 10 sessions of active treatment, no statistically significant differences were evident between the treatment groups in patterns of treatment attendance. Therefore, according to this index of acceptability, it is reasonable to suggest that people in the CAC treatment found this mode of delivery as acceptable as a therapist-delivered alternative. In a recent review of the acceptability of computerized CBT for depression [17], mean percentage dropout over treatment (ranging from 1 to 33 sessions) was 32% (SD 16.52, range 0%-75%). Take-up rates of computerized treatment reported in the same review ranged from 3% to 25%, although it was likely that these rates also reflected reluctance to enter the trial, not just participation in computerized CBT [17]. Studies of face-to-face CBT for depression have reported dropout rates of up to 38%, with 27%-30% dropout reported in medication trials of antidepressants [17]. These rates are comparable with those reported in this study. 

### Therapeutic Alliance

Results relating to the second criterion of acceptability, therapeutic alliance, also suggested equivalence in outcomes between therapist-delivered and CAC treatments, and, for session 1, a BI. Participants rated therapeutic bond, confidence in therapy, ability to direct therapy, and client openness highly across the treatment conditions at sessions 1 (all conditions), 5, and 10 (therapist-delivered and CAC treatments). It is of note that Client Initiative was rated significantly higher by participants in the CAC condition at session 5, relative to the therapist-delivered condition. Although this difference had disappeared by session 10, it suggests increased empowerment and enhanced problem-solving skills potentially associated with the “self-help” nature of computer-based treatment. As a similar result regarding Client Initiative was obtained for the BI relative to the therapist-delivered alternative after session 1, similar alliance mechanisms may be operating in the BI and CAC conditions among this comorbid group. Over the course of treatment, total alliance scores increased by 2 points from session 1 to session 10, with no significant differences evident between the therapist-delivered and CAC treatment groups. In addition, therapeutic alliance scores (total and subscale scores) across all time points were not predicted by treatment allocation, nor by any of the models tested in the regression analysis. 

No previous study has reported on therapeutic alliance among people completing therapist-delivered versus CAC treatments for depression and AOD use problems; however, studies of computerized CBT for other mental health conditions have generally reported patient satisfaction and acceptability of this mode of delivery [17]. For example, in a large-scale randomized controlled trial conducted in the United Kingdom, Proudfoot and colleagues [27] compared an eight-session computerized CBT with treatment as usual among 274 people with depression or anxiety-related conditions. Average satisfaction with treatment was over one and a half times higher in the computer group relative to controls who received treatment as usual [27]. Attrition rates were comparable with those encountered in face-to-face therapies, with around 35% of computer participants not completing their full complement of sessions. 

The real-world implications of these results are potentially important. Namely, a group of people with moderate and severe levels of comorbid depression and AOD use problems, who are challenging to engage and retain, and are regarded as complicated to treat effectively [28-30], participated in computer-based treatment with reduced therapist input over 10 sessions with equivalent dedication and attachment to a face-to-face therapy. This engagement occurred with a computer-delivered program, requiring only 12.5 minutes per session of generalist therapist input over the treatment period [16]. Our previous research has also indicated that CAC treatment was as effective in improving depression, AOD use, and functioning outcomes as the therapist-delivered equivalent [16]. 

Early alliance ratings (session 5 or earlier) have generally demonstrated higher predictive value, in terms of symptom reduction and other posttreatment outcomes, than later-therapy alliance and/or average alliance [31-33]. Although this was generally not true for our sample, changes in alcohol use were associated with higher levels of Client Initiative across therapy. Individuals in the BI (after session 1) and CAC interventions (after session 5 and overall) rated Client Initiative significantly and consistently higher than the therapist-delivered treatment. The implication of these results for early alliance is that treatments requiring less therapist contact may be more effective at enhancing self-directedness and responsibility for directing treatment and change, and in this context this may have translated into improved alcohol use outcomes. Therapists involved in ongoing contact with clients may need to attend more to encouraging Client Initiative for change early in treatment, with this taking precedence over technical interventions in the beginning of therapy [34]. It may also be that more time spent on motivational approaches is important in this context [32]. 

### Limitations

There are several limitations to this study, not the least of which is the small sample size and participant attrition in relation to therapist alliance ratings. In substituting data for participants who did not complete the session 5 and session 10 ARM ratings, we assumed no change, when alliance may have deteriorated. This may have inflated the improvement observed in therapeutic alliance over the treatment period reported in relation to Table 3. Further, in predicting therapeutic alliance, and in examining the associations between treatment allocation and therapeutic alliance, power to detect differences between therapist-delivered and CAC groups was low to moderate (range 0.1-0.63). Looking at the data, actual differences between these treatment groups in alliance measures was 0.08-0.15 for Bond, Confidence, and Openness, with the largest differences in alliance observed for Client Initiative (0.59) and the total alliance score (1.28) in favor of higher scores for CAC participants. Therefore, we remain cautiously confident in our assertion that there was little notable difference in alliance ratings and acceptability of CAC versus therapist-delivered treatments offered in our study. However, replication is required to further explore these results. In addition, the extra benefit of the brief check-in sessions conducted with all CAC participants cannot be quantified in this study and may well have influenced the equivalence in therapist-delivered versus CAC outcomes. However, significantly reduced therapist time was used in the CAC condition, and the content of this interaction was generalized and could reasonably be applied by professionals working in mental health, AOD, and primary care settings [16]. Previous computer experience was not assessed among the CAC participants, nor was preference for a particular mode of treatment delivery. These variables may have affected on the results. It is also possible that the self-referral nature of study recruitment attracted and retained participants with high motivation to attend and complete treatment, manifesting in a high propensity for strong alliance. Results may be different with a less-motivated sample, although a reasonable proportion of participants did report being in the precontemplative stage of change for their AOD use. Anonymous therapeutic alliance data collection did not allow the therapist to monitor completion of the therapeutic alliance measure. A better monitoring system involving the administrative staff might be more successful in encouraging participants to complete the forms in further studies. Kaltenthaler et al [17] suggested that several other components were important in considering acceptability of treatment in this context. These include reasons for dropout, patient satisfaction questionnaires, and expectations of therapy. These domains were not measured in the current study, and it remains important for future research to include measures of these issues. Notwithstanding these limitations, the results support the acceptability of computerized CBT treatments for people with depression and AOD use comorbidity. 

### Summary and Conclusions

No previous research has examined the acceptability and therapeutic alliance of CAC therapy among a group with comorbid depression and AOD use relative to a BI or therapist equivalent, nor with a sample reporting severe levels of depression at baseline and concurrent heavy use of alcohol or cannabis. The results indicate that people with this comorbidity find CAC treatment as acceptable, in terms of treatment dropout and therapeutic alliance, as an equivalent therapist-delivered treatment program. This robust finding was demonstrated across a range of potentially confounding demographic and symptom domains. Rates of dropout in both treatment modalities were equivalent to other treatment trials among people with depression, and among those participating in trials of CBT, despite the study population having current and severe comorbidity and being stereotypically difficult to attract, retain, and treat effectively. 

The extent to which client characteristics and alliance may work together to moderate posttreatment outcomes still needs to be determined. Symptom and functioning outcomes of CAC versus therapist-delivered treatment have been reported elsewhere [16]; however, short-term change in depression, alcohol, or cannabis use in this study was not associated with changes in therapeutic alliance, with the exception of Client Initiative and hazardous alcohol use. Both the BI and CAC interventions, which required less therapist contact, were associated with significantly elevated Client Initiative relative to therapist-delivered treatment. This suggests that initiative may be an important element in nontherapist-directed change.

The promising results regarding the acceptability of CAC treatment to a complex comorbid group are important, considering that the computer-delivered intervention used an average of 12.5 minutes face-to-face clinician time per session compared with approximately 1 hour of face-to-face therapy among the therapist-delivered equivalents. In Australia, 67% of people with mental health problems do not access treatment for their conditions [35,36]. Together with evidence that the majority of people prefer to manage on their own, including a substantial proportion with comorbid conditions [37], the potential for computer-based self-help treatments is promising. For people with comorbid depression and AOD use problems in particular, who report increasing difficulties accessing treatments when sought, computer-based therapy means easier access to evidence-based treatment [38]. Computer-based therapy could result in more people seeking treatment for their condition, or receiving treatment in an earlier phase of their disorder. Potentially, this could prevent conditions such as alcohol misuse, other problematic substance use, and depression from becoming more chronic and disabling, relieving the disease burden on mental health services and the community [38]. The self-help nature of the BI and CAC interventions offered in this study was associated with superior Client Initiative to face-to-face treatment, and may better empower people to become more actively involved in their own health care. Clinician contact in the computer condition was generic in nature, and could potentially be delivered via telephone, email or other modalities rather than face to face. In addition, this generic contact could be provided by many generalist health and primary care professionals, not necessarily those with specialist psychological or comorbidity-specific training. Clearly, access to BIs and computer-based health care stands to be a key driver of improved mental health and general health outcomes for this highly comorbid group within the community.

## Abbreviations

ANOVA: analysis of variance
AOD: alcohol/other drug
ARM: Agnew-Davies Relationship Measure
BDI-II: Beck Depression Inventory II
BHS: Beck Hopelessness Scale
BI: brief intervention
CAC: clinician-assisted computer-based
CBT: cognitive behavior therapy
MI: motivational interviewing
OTI: Opiate Treatment Index
